# Extrapolation in the analysis of lung aeration by computed tomography: a validation study

**DOI:** 10.1186/cc10563

**Published:** 2011-11-23

**Authors:** Andreas W Reske, Anna Rau, Alexander P Reske, Manja Koziol, Beate Gottwald, Michaele Alef, Jean-Claude Ionita, Peter M Spieth, Pierre Hepp, Matthias Seiwerts, Alessandro Beda, Silvia Born, Gerik Scheuermann, Marcelo BP Amato, Hermann Wrigge

**Affiliations:** 1 Department of Anesthesiology and Intensive Care Medicine, University Hospital Leipzig, Liebigstrasse 20, D-04103 Leipzig, Germany; 2 Pulmonary Engineering Group, Department of Anesthesiology and Intensive Care Medicine, University Hospital Carl Gustav Carus, Fetscherstrasse 74, D-01307 Dresden, Germany; 3 Large Animal Clinic for Surgery, Faculty of Veterinary Medicine, University of Leipzig, An den Tierkliniken 21, D-04103 Leipzig, Germany; 4 Department of Small Animal Medicine, Faculty of Veterinary Medicine, University of Leipzig, An den Tierkliniken 23, D-04103 Leipzig, Germany; 5 Department of Trauma and Reconstructive Surgery, University Hospital Leipzig, Liebigstrasse 20, D-04103 Leipzig, Germany; 6 Department of Diagnostic and Interventional Radiology, University Hospital Leipzig, Liebigstrasse 20, D-04103 Leipzig, Germany; 7 Postgraduate Electrical Engineering Program, Institute of Technology, Federal University of Pará, Campus Universitário do Guamá, Guamá, 66075-110 - Belém, Pará, Brazil; 8 Innovation Center Computer Assisted Surgery (ICCAS), Faculty of Medicine, University of Leipzig, Semmelweisstrasse 14, D-04103 Leipzig, Germany; 9 Institute of Computer Science, Faculty of Mathematics and Computer Science, University of Leipzig, Johannisgasse 26, D-04103 Leipzig, Germany; 10 Cardio-Pulmonary Department, Pulmonary Divison, Hospital das Clínicas, University of São Paulo Medical School, Av. Dr Arnaldo 455 (room 2206, 2 nd floor), São Paulo 01246-903, Brazil

## Abstract

**Introduction:**

Computed tomography (CT) is considered the gold standard for quantification of global or regional lung aeration and lung mass. Quantitative CT, however, involves the exposure to ionizing radiation and requires manual image processing. We recently evaluated an extrapolation method which calculates quantitative CT parameters characterizing the entire lung from only 10 reference CT-slices thereby reducing radiation exposure and analysis time. We hypothesized that this extrapolation method could be further validated using CT-data from pigs and sheep, which have a different thoracic anatomy.

**Methods:**

We quantified volume and mass of the total lung and differently aerated lung compartments in 168 ovine and 55 porcine whole-lung CTs covering lung conditions from normal to gross deaeration. Extrapolated volume and mass parameters were compared to the respective values obtained by whole-lung analysis. We also tested the accuracy of extrapolation for all possible numbers of CT slices between 15 and 5. Bias and limits of agreement (LOA) were analyzed by the Bland-Altman method.

**Results:**

For extrapolation from 10 reference slices, bias (LOA) for the total lung volume and mass of sheep were 18.4 (-57.2 to 94.0) ml and 4.2 (-21.8 to 30.2) grams, respectively. The corresponding bias (LOA) values for pigs were 5.1 (-55.2 to 65.3) ml and 1.6 (-32.9 to 36.2) grams, respectively. All bias values for differently aerated lung compartments were below 1% of the total lung volume or mass and the LOA never exceeded ± 2.5%. Bias values diverged from zero and the LOA became considerably wider when less than 10 reference slices were used.

**Conclusions:**

The extrapolation method appears robust against variations in thoracic anatomy, which further supports its accuracy and potential usefulness for clinical and experimental application of quantitative CT.

## Introduction

Important insights and key hypotheses for understanding the pathophysiology and treatment of acute respiratory failure were obtained by computed tomography (CT)-based imaging and quantification of global or regional lung aeration. Currently, CT is considered the gold standard for this purpose [1-15]. Quantitative analysis of CT, however, has important practical limitations. The exposure to ionizing radiation limits clinical application and the long time required for manual image processing significantly complicates the analysis of CT data. Particularly in animal research, whole-lung CT is often performed repeatedly during different lung conditions, which results in huge numbers of CT images to be processed manually. Such an analysis can easily require several hours for a single whole-lung CT scan, consuming considerable research resources.

Extrapolation from reference CT-slices is an option to reduce radiation exposure and to simplify the calculation of quantitative CT parameters characterizing the entire lung [16,17]. We recently evaluated this extrapolation method for human patients [18].

In the present study, we used pulmonary CT scans of pigs and sheep, whose thoracic shape and intrathoracic anatomy differ significantly from humans, for further validation of the accuracy of the extrapolation technique. We hypothesized that the extrapolation method is robust against variations in thoracic anatomy which would further support its accuracy and usefulness for clinical and experimental application of quantitative CT.

## Materials and methods

All CT scans covering the entire lungs of Merino sheep or Landrace pigs available in the CT database of our research group were identified and retrospectively analyzed in this work. This paper focuses solely on the validation of a method for obtaining quantitative CT analyses characterizing the entire lung by extrapolation. Therefore, hemodynamic and respiratory variables are not reported.

All experimental protocols were approved by the animal ethics committees of the University of Leipzig (TVV18/06, TVV17/08) and the University of Sao Paulo. The handling of the animals complied with the NIH guidelines for animal use [19]. Adequate anesthesia was induced and maintained by bolus injections and subsequent continuous infusion of ketamine, propofol, midazolam and/or fentanyl in pigs, and xylazine, midazolam, propofol and/or sufentanil in sheep.

**Figure 1:**
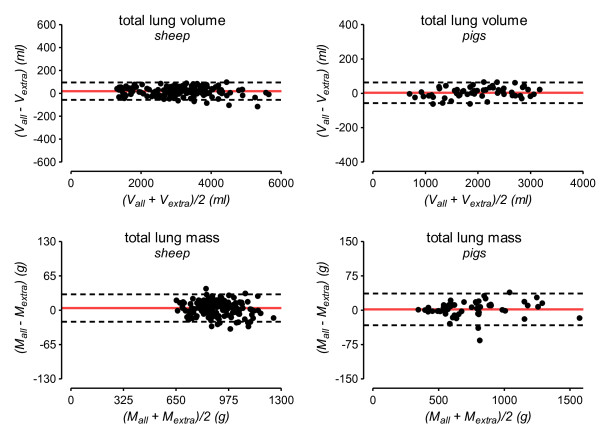
**Bland-Altman plots for the agreement between extrapolation and whole-lung analysis**. The solid red line indicates the mean difference between whole-lung CT analysis and extrapolation (bias) and the dashed red lines correspond to the 95% limits of agreement. To cover a broad range of lung volume and mass for each species, CT data from normal and opacified lungs were pooled for this analysis. g, grams; ml, milliliters; V_all_/M_all_, lung volume or mass calculated from all CT-slices covering the entire lung; V_extra_/M_extra_, lung volume or mass obtained by extrapolation.

**Figure 2:**
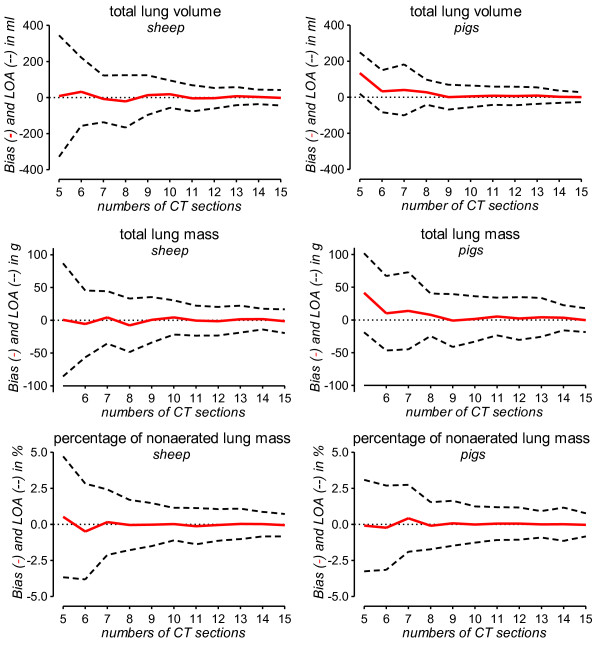
**Accuracy of extrapolation for different numbers of reference CT slices**. The accuracy of extrapolation was assessed by Bland-Altman analysis for different numbers of reference CT slices, which are plotted on the x-axis. To cover a broad range of lung volumes and masses for this analysis, CT data from normal and opacified lungs were pooled for each species. The solid red line indicates the mean difference between whole-lung CT analysis and extrapolation (bias) for each number of reference CT slices used for extrapolation. The dashed lines indicate the 95% limits of agreement (LOA). In addition to total lung volume and mass, the nonaerated lung compartment was chosen for presentation because of its pathophysiological relevance.

The whole-lung CT scans available covered a broad range of lung conditions from normal aeration to gross deaeration and were separated by two independent observers into two groups for each species: normal lungs (normal) and lungs with opacifications (opacified). Only CT findings were considered for group allocation: animals with lung opacifications other than small, localized dorsal atelectasis were included in the respective opacified group. Data characterizing lung conditions and the accuracy of extrapolation are presented separately for normal and opacified groups. To cover a broad range of lung aeration, both groups were pooled for each species for the analyses shown in Figures 1 and 2.

### Pig experiments

Fifty-five whole-lung CT scans of mechanically ventilated pigs (range of body weight 27 to 33 kg) were considered in this study. Fourteen normal pulmonary CT scans of pigs which underwent diagnostic CT to exclude pneumonic infiltrates before other experiments were studied. A recruitment maneuver was performed before these CTs to reinflate atelectasis potentially obscuring small infiltrates. Forty-one porcine CT scans showing diffuse pulmonary opacifications resulting from experimental lung injury (repeated lung lavage with normal saline [13,20]) were analyzed.

### Sheep experiments

One hundred sixty-eight whole-lung CT scans of mechanically ventilated sheep (range of body weight 46 to 70 kg) were indentified in our database. Sixty-six CT scans of normal lungs of sheep which underwent diagnostic CT for the exclusion of pneumonic infiltrates were studied. A recruitment maneuver was performed before these CTs to reinflate atelectasis potentially obscuring small infiltrates. We also studied 102 lung CT scans of sheep with bilateral focal opacifications (dependent atelectasis) which developed during mechanical ventilation with pure oxygen. For 58 of the ovine CTs, a corresponding CT performed before and after an increase of airway pressure was available. These 58 pairs of consecutive CT scans reflecting substantially different lung conditions were analyzed in order to explore whether the extrapolation method can also be used to assess intra-individual changes of quantitative CT parameters.

### Quantitative CT analysis and extrapolation method

Images used had been generated by two different models of multi-slice CT scanners, either a Somatom Volume Zoom (120-kV tube voltage, 165-mA tube current, 4 × 2.5-mm collimation; Siemens, Erlangen, Germany) or a Philips MX8000 IDT 16 (120-kV tube voltage, 170-mA tube current, 16 × 1.5-mm collimation; Philips Medical Systems, Hamburg, Germany). All CT images were reconstructed with standard reconstruction filters ("B35 f" on the Siemens and "B" on the Philips scanner) and slice thicknesses between 5 and 10 mm [21,22]. All quantitative analyses of CT data were performed at the University Hospital Leipzig. Six members of the research group at Leipzig University Hospital performed the manual image segmentations, while three other authors monitored the segmentations and performed the extrapolations. The distribution of pulmonary opacifications was classified by two independent observers considering all CT slices available [4]. The Osiris software (University Hospital Geneva, Switzerland) was used for manual segmentation of the lung parenchyma. Identical to our previous studies, major pulmonary vessels, trachea and main bronchi were excluded [18,22,23]. Total lung volume (V_total_) and mass (M_total_) were calculated voxel-by-voxel from all lung voxels within the -1,000 to +100 Hounsfield units (HU) range. Volumes (%V) and masses (%M) of differently aerated lung compartments were calculated as percentage of V_total _or M_total_, respectively. The following HU-ranges were used to define differently aerated lung compartments: nonaerated (%V_non_,%M_non_), -100 to +100 HU; poorly aerated (%V_poor_,%M_poor_), -101 to -500 HU; normally aerated (%V_norm_,%M_norm_), -501 to -900 HU; hyperaerated (%V_hyper_,%M_hyper_), -901 to -1000 HU [2,3,18,23].

In addition to the analysis of all CT slices covering the entire lung (whole-lung analysis), the calculation of volumes and masses characterizing the entire lung was performed by extrapolation from only 10 reference CT slices, as previously described [16-18]. Briefly, the most cranial and most caudal CT slices displaying lung tissue and eight equidistant CT slices between them were selected. The extrapolation of quantitative CT data resulting from these 10 reference slices to the entire lung was performed as follows: mean values of each pair of consecutive slices were divided by nominal slice thickness and multiplied by the interval between the slice positions. All resulting products were summed up and a correction term was added [17,18]. All steps of the extrapolation procedure except for the identification of the most cranial and most caudal CT slices were performed automatically by dedicated software. Using the same approach, we also tested the extrapolation method for different slice thicknesses as well as for each possible number of reference CT slices between 15 and 5 slices.

Examples for radiation doses were calculated using the CT-Expo software (Department of Diagnostic Radiology, Hannover Medical School, Hannover, Germany).

### Statistics

Results are reported as median and range (minimum and maximum values). The agreement between extrapolation and whole-lung CT analysis was assessed according to Bland and Altman and is reported as bias and limits of agreement [24,25]. The GraphPad Prism 5 software was used for statistical analyses (GraphPad Software, La Jolla, CA, USA).

## Results

The median height (cranio-caudal distance) was 278 (222 to 320) mm for normal sheep lungs and 270 (218 to 308) mm for normal pig lungs. The median number of CT slices covering the entire lung of sheep was 24 (20 to 30) for 10 mm slice thickness and 55 (44 to 64) for 5 mm slices. The median number of CT slices covering the entire lung of pigs was 34 (25 to 41) for 7.5 mm and 54 (48 to 62) for 5 mm slice thickness.

Values characterizing lung volumes and masses calculated from whole-lung analysis are given in Table 1. The classification as normal or opacified is supported by the respective amounts of nonaerated lung in these groups. Animals with opacified lungs had relevant (up to 60%) amounts of nonaerated lung (Table 1).

**Table 1:** Lung volumes and masses quantified by whole-lung CT analysis

	Normal lung CT scans		Lung CT scans with opacification	
	sheep (*n *= 66)	pigs (*n *= 14)	sheep (*n *= 102)	pigs (*n *= 41)
V_total _(ml)	3,748 (2,245 to 5,638)	2,308 (1,114 to 3,061)	2,827 (1,338 to 4,252)	1,868 (698 to 3,184)
V_non _(%)	0.5 (0.0 to 0.8)	0.1 (0.0 to 1.2)	3.9 (0.3 to 32.2)	3.6 (0.0 to 43.5)
V_poor _(%)	3.4 (1.4 to 10.7)	2.3 (0.8 to 10.7)	10.7 (3.5 to 46.7)	27.8 (4.3 to 59.1)
V_normal _(%)	93.7 (88.4 to 97.5)	94.7 (88.9 to 98.2)	82.4 (33.5 to 94.1)	68.5 (22.5 to 95.5)
V_hyper _(%)	1.4 (0.1 to 5.6)	1.8 (0.3 to 4.9)	0.7 (0.0 to 8.0)	0.1 (0.0 to 2.0)
M_total _(g)	891 (694 to 1,116)	505 (397 to 703)	888 (660 to 1,246)	795 (346 to 1,564)
M_non _(%)	1.9 (0.2 to 2.8)	0.3 (0.0 to 5.3)	10.6 (1.3 to 56.0)	10.1 (0.0 to 58.5)
M_poor _(%)	8.7 (4.7 to 18.8)	5.8 (2.5 to 17.8)	22.2 (9.2 to 60.2)	35.3 (7.4 to 67.1)
M_normal _(%)	88.3 (78.9 to 93.2)	90.3 (77.5 to 96.9)	61.3 (18.5 to 87.0)	48.1 (10.2 to 92.5)
M_hyper _(%)	0.5 (0.0 to 2.2)	0.5 (0.1 to 2.4)	0.1 (0.0 to 3.2)	0.0 (0.0 to 0.6)
V_gas _(ml)	2,855 (1,479 to 4,556)	1,761 (717 to 2,523)	1,904 (482 to 3,133)	1,085 (196 to 2,169)
V_gas _(%)	76.4 (65.9 to 80.8)	76.7 (64.4 to 82.4)	66.9 (32.8 to 78.0)	55.4 (24.8 to 73.2)

Data are given as median (range).

CT, computed tomography; V_total_/M_total_, total lung volume or mass. The following volumes and masses of differently aerated lung compartments were calculated as percentage of V_total _or M_total_, respectively: V_non_/M_non _, nonaerated volume or mass; V_poor_/M_poor_, poorly aerated volume or mass; V_nomal_/M_normal_, normally aerated volume or mass; V_hyper_/M_hyper_, hyperaerated volume or mass; V_gas_, pulmonary gas volume; ml, milliliters; g, grams.

Bland-Altman plots illustrating the agreement of V_total _and M_total _obtained either by extrapolation from 10 CT slices or by whole-lung analysis are shown in Figure 1.

Absolute bias values between extrapolation from 10 CT slices and the corresponding results of whole-lung CT analysis are given in Table 2. For normal sheep lungs, these bias values corresponded to 1.0% (0.0 to 2.6%) for V_total _and 1.2% (0.0 to 3.6%) for M_total _(percentage of the respective value obtained by whole-lung CT analysis). The corresponding values were 1.2% (0.0 to 4.2%) and 1.2% (0.0 to 4.8%) for V_total _and M_total_, respectively, for opacified sheep lungs. For normal porcine lungs, the corresponding bias values were 0.9% (0.1 to 2.3%) for V_total _and 0.5% (0.0 to 3.6%) for M_total_. Finally, for porcine lungs with opacifications these bias values were 1.1% (0.1 to 5.6%) and 1.5% (0.1 to 8.5%) for V_total _and M_total_, respectively.

**Table 2:** Agreement between extrapolation from 10 CT slices and whole-lung analysis

	Normal lung CT scans		Lung CT scans with opacification	
	sheep (*n *= 66)	pigs (*n *= 14)	sheep (*n *= 102)	pigs (*n *= 41)
V_total _(ml)	19.6 (-64.6 to 103.8)	14.5 (-26.7 to 55.8)	17.6 (-52.2 to 87.5)	1.8 (-62.9 to 66.5)
V_non _(%)	0.0 (-0.2 to 0.2)	0.0 (-0.2 to 0.2)	0.0 (-0.7 to 0.6)	0.0 (-1.2 to 1.2)
V_poor _(%)	0.0 (-0.5 to 0.5)	0.1 (-0.5 to 0.7)	-0.3 (-1.8 to 1.2)	0.1 (-1.9 to 2.0)
V_normal _(%)	0.1 (-0.8 to 0.9)	-0.1 (-0.5 to 0.4)	0.3 (-1.4 to 2.0)	-0.1 (-2.1 to 1.9)
V_hyper _(%)	-0.1 (-0.7 to 0.6)	0.0 (-0.7 to 0.7)	0.0 (-0.4 to 0.5)	0.0 (-0.2 to 0.1)
M_total _(g)	6.1 (-19.6 to 31.9)	3.3 (-11.6 to 18.2)	2.9 (-23.1 to 29.0)	1.0 (-38.1 to 40.2)
M_non _(%)	0.1 (-0.6 to 0.7)	-0.1 (-0.9 to 0.7)	0.0 (-1.4 to 1.3)	0.0 (-1.4 to 1.4)
M_poor _(%)	-0.1 (-1.1 to 1.0)	0.3 (-1.0 to 1.6)	-0.3 (-2.1 to 1.4)	0.1 (-2.0 to 2.2)
M_normal _(%)	0.0 (-1.4 to 1.4)	-0.2 (-1.4 to 1.0)	0.4 (-1.4 to 2.2)	-0.1 (-2.0 to 1.8)
M_hyper_(%)	0.0 (-0.3 to 0.2)	0.0 (-0.3 to 0.3)	0.0 (-0.1 to 0.1)	0.0 (0.0 to 0.0)
V_gas _(ml)	13.5 (-51.5 to 78.4)	11.2 (-25.4 to 47.8)	14.7 (-39.8 to 69.2)	0.8 (-37.7 to 39.3)
V_gas _(%)	0.0 (-0.4 to 0.4)	0.0 (-0.5 to 0.6)	0.2 (-0.7 to 1.1)	-0.1 (1.2 to 1.1)

Data are bias (95% limits of agreement) from the Bland-Altman analysis of the agreement between extrapolation from 10 CT slices and analysis of all CT slices covering the entire lung.

CT, computed tomography; g, grams; ml, milliliters; V_gas_, pulmonary gas volume; V_hyper_/M_hyper_, hyperaerated volume or mass; V_nomal_/M_normal_, normally aerated volume or mass; V_non_/M_non_, nonaerated volume or mass; V_poor_/M_poor_, poorly aerated volume or mass; V_total_/M_total_, total lung volume or mass. Volumes and masses of differently aerated lung compartments were calculated as percentage of V_total _or M_total_, respectively.

The bias between methods for volume and mass of differently aerated lung compartments never exceeded 0.5% and the respective limits of agreement were below 2.5% of V_total _or M_total_, respectively (Table 2).

The accuracy of extrapolation for varying numbers (15 to 5) of CT slices is illustrated in Figure 2. For extrapolation from 10 or more CT slices, the bias values for V_total_, M_total _and%M_non _were very close to 0 and the limits of agreement were narrow. When less than 10 CT slices were used for extrapolation, bias values started to diverge from 0 and the 95% limits of agreement started to become considerably wider (Figure 2).

Variations in CT slice thickness between 5 and 10 mm did not have an effect on the accuracy of extrapolation (Table 3).

**Table 3:** Accuracy of extrapolation for CT sections with different slice thickness

	Sheep			Pig	
thickness	10 mm	6 mm	5 mm	7.5 mm	5 mm
*n *=	35	12	121	38	17
V_total _(ml)	16.0 (-60.7 to 92.8)	25.1 (48.0 to 98.2)	18.4 (-57.5 to 94.4)	3.0 (-57.0 to 63.0)	9.6 (-52.2 to 71.4)
V_non _(%)	-0.1 (-0.9 to 0.8)	-0.1 (-0.4 to 0.3)	0.0 (-0.4 to 0.4)	0.0 (-1.2 to 1.2)	0.0 (-0.4 to 0.5)
V_poor _(%)	-0.5 (-2.6 to 1.6)	-0.2 (-0.8 to 0.3)	-0.1 (-0.9 to 0.7)	0.2 (-1.6 to 2.0)	-0.2 (-1.5 to 1.2)
V_normal _(%)	0.6 (-1.8 to 3.0)	0.3 (-0.4 to 1.0)	0.1 (-0.9 to 1.1)	-0.2 (2.0 to 1.7)	-0.2 (1.2 to 1.5)
V_hyper _(%)	0.0 (-0.2 to 0.3)	0.0 (-0.1 to 0.1)	0.0 (-0.6 to 0.6)	0.0 (-0.1 to 0.1)	-0.1 (-0.7 to 0.6)
M_total _(g)	3.9 (-23.4 to 31.2)	4.7 (-19.3 to 28.8)	4.2 (-21.8 to 30.3)	1.9 (-32.7 to 36.5)	1.0 (-34.4 to 36.5)
M_non _(%)	-0.1 (-1.5 to 1.4)	-0.2 (1.1 to 0.7)	0.1 (1.0 to 1.1)	-0.1 (-1.3 to 1.2)	0.1 (-1.2 to 1.4)
M_poor _(%)	-0.5 (-2.5 to 1.6)	-0.4 (-1.4 to 0.6)	0.1 (-1.5 to 1.2)	0.2 (-1.7 to 2.2)	-0.1 (-1.8 to 1.7)
M_normal _(%)	0.5 (-1.5 to 2.5)	-0.6 (-0.8 to 1.9)	0.1 (-1.5 to 1.7)	-0.2 (-2.0 to 1.7)	0.0 (-1.6 to 1.5)
M_hyper _(%)	0.0 (-0.1 to 0.1)	0.0 (0.0 to 0.0)	0.0 (-0.2 to 0.2)	0.0 (-0.1 to 0.1)	0.0 (-0.3 to 0.3)
V_gas _(ml)	12.1 (-42.8 to 67.0)	20.4 (-32.6 to 73.4)	14.2 (-46.3 to 74.7)	1.2 (-37.3 to 39.6)	8.5 (-30.2 to 47.2)

Data are bias (95% limits of agreement) from the Bland-Altman analysis of the agreement between extrapolation from 10 CT slices and analysis of all CT slices covering the entire lung. Columns present this data for subgroups of CT image series reconstructed with a certain slice thickness. CT, computed tomography; g, grams; ml, milliliters; n, number of data sets; V_gas_, pulmonary gas volume; V_hyper_/M_hyper_, hyperaerated volume or mass; V_nomal_/M_normal_, normally aerated volume or mass; V_non_/M_non_, nonaerated volume or mass; V_poor_/M_poor_, poorly aerated volume or mass; V_total_/M_total_, total lung volume or mass. Volumes and masses of differently aerated lung compartments were calculated as percentage of V_total _or M_total_, respectively.

**Figure 3:**
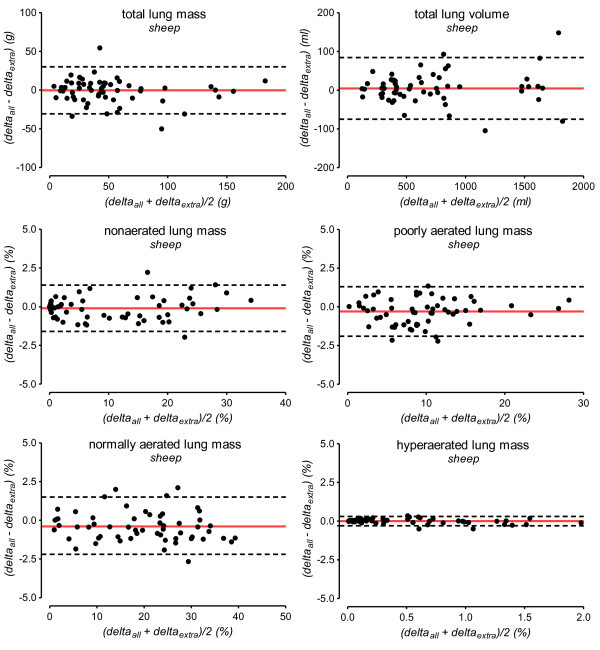
**Accuracy of extrapolation for assessing changes of lung volume and masses between consecutive CTs**. The accuracy of the extrapolation method for assessing changes (delta) of lung volume and masses between consecutive CTs was analyzed by the Bland-Altman method in 58 pairs of consecutive CTs. The solid red line indicates the mean difference between whole-lung CT analysis and extrapolation (bias). The dashed lines indicate the 95% limits of agreement. Delta_all_, changes of quantitative CT parameters (total lung volume and mass, mass of differently aerated lung compartments) between two consecutive CTs assessed by whole-lung analysis; Delta_extra _, changes of parameters between two consecutive CTs calculated by extrapolation; g, grams; ml, milliliters.

Results on the accuracy of the extrapolation method for detecting intra-individual changes between two consecutive CTs are shown in Table 4 and Figure 3.

**Table 4:** Intra-individual changes between 58 pairs of consecutive CT scans in sheep

	Changes between CTs (whole-lung analysis)	Bias (limits of agreement)
V_total _(ml)	551 (121 to 1863)	6.5 (-72.5 to 85.5)
V_non _(%)	2.6 (0.0 to 11.6)	-0.1 (-0.5 to 0.4)
V_poor _(%)	4.8 (0.3 to 20.7)	-0.2 (-1.1 to 0.8)
V_norm _(%)	8.5 (0.0 to 22.2)	-0.2 (-1.4 to 0.9)
V_hyper _(%)	0.5 (0.0 to 4.5)	0.0 (-0.8 to 0.8)
V_gas _(%)	6.4 (1.6 to 16.0)	-0.1 (-0.9 to 0.7)
M_total _(g)*	43 (0 to 189)	-1.5 (-33.0 to 30.0)
M_non _(%)	6.7 (0.0 to 34.4)	-0.1 (-1.6 to 1.4)
M_poor _(%)	8.7 (0.2 to 28.4)	-0.3 (-2.0 to 1.3)
M_normal _(%)	21.0 (0.7 to 38.7)	-0.4 (-2.3 to 1.5)
M_hyper _(%)	0.2 (0.0 to 1.9)	0.0 (-0.3 to 0.3)

Data are median (range) for changes of lung volume and masses between two consecutive CTs given in the left column. Bias (95% limits of agreement) from the Bland-Altman analysis of the agreement of changes calculated by extrapolation from 10 CT slices or whole-lung analysis are given in the right column. CT, computed tomography; g, grams; M_hyper_, hyperaerated volume or mass; ml, milliliters; M_non_, nonaerated volume or mass; M_normal_, normally aerated volume or mass; M_poor_, poorly aerated volume or mass; V_gas_, pulmonary gas volume; V_total_/M_total_, total lung volume or mass. Volumes and masses of differently aerated lung compartments were calculated as percentage of V_total _or M_total_, respectively. *The sheep studied had gross atelectasis of otherwise normal lungs; the change in airway pressure therefore resulted in large changes in lung volume but only small changes in total lung mass.

## Discussion

Our study demonstrates the excellent accuracy of the extrapolation method for calculation of parameters characterizing the entire lung from only 10 reference CT slices. The extrapolation method was also very accurate in detecting changes between two consecutive CTs. Our results confirm a previous report on the accuracy of such an extrapolation method in humans [18] and demonstrate that the extrapolation method is robust against variations in thoracic anatomy. The bias between extrapolation and whole-lung CT analysis increased progressively when less than 10 reference CT slices were used for extrapolation.

Quantification of lung aeration by CT has become an important research tool for studying normal and pathological aspects of lung aeration as well as the effects of mechanical ventilation on lung aeration. Particularly in the fields of emphysema and acute lung injury, quantitative CT has become a central tool for both clinical and experimental research [1-15,17,23,26-30].

In contrast to emphysema, which can already be quantified automatically in CT images, manual interaction is often required for analysis of CT images with opacifications. Lung opacification occurring in diseases such as acute lung injury frequently have tissue densities (and thus CT numbers) close to that of the soft tissues of the thoracic wall, mediastinum or diaphragm and are thus difficult to differentiate from these non-pulmonary tissues. Although initial experiences with automatic segmentation techniques of opacified lungs have been reported, none of these techniques is already available for broad experimental or even clinical use [29-31]. Consequently, the time required for manual analysis of a single whole-lung CT (median 55 slices of 5 mm thickness) can easily exceed five hours. The potential of the extrapolation method for saving time and research resources becomes obvious when considering that the work required for manual interaction can be decreased by up to 80% when only 10 reference CT slices are analyzed. Extrapolation of adipose tissue volumes or pulmonary gas volumes has been applied by other investigators in order to limit radiation in quantitative CT studies in patients [16,17]. Our current results support this method of calculation and further underline that extrapolation is an option to simplify quantitative CT analysis. In the experimental setting, full spiral CT scans may be performed to acquire maximum information, but for the purpose of gas and tissue quantification, analysis can be limited to 10 scans. True limitation of radiation, however, can only be achieved if 10 separate single slices of a certain thickness are "prospectively" planned and scanned one by one [17], which differs completely from spiral CT of the whole chest. Calculation of examples for effective radiation doses (for the Philips scanner) indicates that the effective radiation dose can be decreased by approximately 50% from 3.8 mSv (spiral CT) to 2 mSv (10 single slices).

In our opinion, several reasons preclude the generalized recommendation to use less than 10 reference CT slices. As explained by Gattinoni *et al*. in a recent editorial, 10 slices with 10 mm thickness cover about 40% of the lung tissue whereas 10 slices with 5 mm thickness contain only about 20% of the lung [26]. If the number or thickness of reference slices is decreased too much, the density information available for extrapolation and consequently the accuracy of extrapolation decreases. As illustrated in Figure 2, analyzing 10 reference slices seems to be a reasonable compromise: bias values diverged from zero and the limits of agreement became considerably wider when less than 10 reference slices were used. As pointed out in our previous study in human patients, the adequate number of reference CT slices required for accurate extrapolation of quantitative CT results varies with the study purpose [18]. While quantitative assessment of differently aerated lung compartments seems possible using less than 10 reference slices, we suggest using a minimum of 10 slices, especially whenever precise quantification of the total lung volume and/or mass is necessary. In the present study, we pooled CT data with slice thickness between 5 and 10 mm in the same analysis because the accuracy of extrapolation did not differ with slice thickness (Table 3). This lack of an effect of slice thickness on the accuracy of extrapolation is in line with our recent study [18]. We intentionally omitted the evaluation of the appropriateness of thinner slices for extrapolation because thin slices can introduce artifacts into quantitative CT analysis [22].

Mostly in animal experiments but also in some clinical situations [2,3,26], whole-lung quantitative CT is performed repeatedly during different lung conditions. Consequently, changes of lung volume, mass or differently aerated lung compartments are common endpoints of such CT studies. We demonstrated that extrapolation enabled accurate quantification of intraindividual changes between consecutive CTs. Therefore, the extrapolation method will also ease analysis of repeated CT scans of the lung.

### Limitations of our study

The principal limitations related to the retrospective study design are acknowledged but appear to be of limited importance to our analyses: The difference between the slice locations of prospectively or retrospectively chosen reference CT slices would differ by only a few millimeters [18]. Given this marginal difference between prospective and retrospective validation of the extrapolation method, we considered it unjustified to perform additional dedicated animal experiments. We could not include animals with a patchy distribution of lung opacifications in our analysis. In a recent study evaluating the extrapolation method in humans, however, we have shown that neither the lung condition nor the distribution of opacifications affected the accuracy of extrapolation [18]. Moreover, common animal models of acute lung injury only rarely lead to real patchy lesions [5,8-10,13,20]. Blinding of investigators involved in the extrapolation procedure to the results of the respective whole-lung analyses was not considered necessary. The potential for investigator bias was significantly limited by the use of dedicated software which, after manual identification of the most cranial and most caudal CT slices, performed all steps of the extrapolation procedure and all calculations automatically.

## Conclusions

The extrapolation method validated in this paper is highly accurate and has the potential to reduce significantly both radiation exposure and the time until the quantitative CT results are available. Our results of CT analyses in pigs and sheep indicate that the extrapolation method is robust against variations in thoracic anatomy, which further supports its accuracy, and potential usefulness for clinical and experimental application of quantitative CT.

## Key messages

• Extrapolation of parameters characterizing the entire lung from only 10 reference CT slices is highly accurate for both analysis of single CTs and the quantification of changes between two consecutive CTs.

• Extrapolation is an option to overcome present limitations of the clinical and experimental application of quantitative CT by reducing manual analysis work and radiation exposure significantly.

• The bias between extrapolation and whole-lung CT analysis increases progressively when less than 10 reference CT slices are used for extrapolation.

## Abbreviations

CT: computed tomography; g: grams; HU: Hounsfield units; kg: kilograms; kV: kilovolts; LOA, 95% limits of agreement: Bland-Altman analysis of agreement; mA: milliamperes; ml: millilitres; mm: millimetres; mSv: millisievert; M_hyper_: mass of the hyperaerated lung compartment,% of total lung mass; M_non_: mass of the nonaerated lung compartment,% of total lung mass; M_normal_: mass of the normally aerated lung compartment,% of total lung mass; M_poor_: mass of the poorly aerated lung compartment,% of total lung mass; M_total_: total lung mass; NIH: National Institutes of Health; V_hyper_: volume of the hyperaerated lung compartment,% of total lung volume; V_non_: volume of the nonaerated lung compartment,% of the total lung volume; V_normal_: volume of the normally aerated lung compartment,% of the total lung volume; V_poor_: volume of the poorly aerated lung compartment,% of the total lung volume; V_totat_: total lung volume.

## Competing interests

The authors declare that they have no competing interests.

## Authors' contributions

MA and JCI were responsible for the data acquisition. SB performed the quantitative CT analysis. AWR, AR and APR planned the study, were responsible for the data acquisition, performed the quantitative CT analysis and were responsible for the statistical analysis and interpretation of the data. MS planned the study, was responsible for the data acquisition and performed the quantitative CT analysis. AB, MBPA and HW planned the study, performed the quantitative CT analysis and were responsible for the statistical analysis and interpretation of the data. PMS planned the study and was responsible for the statistical analysis and interpretation of the data. MK was responsible for the data acquisition, performed the quantitative CT analysis and was responsible for the statistical analysis and interpretation of the data. BG performed the quantitative CT analysis and was responsible for the data acquisition. PH was responsible for the data acquisition and for the statistical analysis and interpretation of data. GS was responsible for the statistical analysis and interpretation of the data and performed the quantitative CT analysis. All authors participated in drafting and critically revising the article. The principal investigators, Dr. Andreas W. Reske, Anna Rau and Hermann Wrigge had full access to the data analyzed in the present study and take full responsibility for the integrity of all of the data and the accuracy of the data analysis. All authors read and approved the final version of the manuscript for publication.
